# Cognitive Mechanisms Underlying Mnemonic Discrimination Across Age And Cultural Groups

**DOI:** 10.1093/geroni/igaf122.1582

**Published:** 2025-12-31

**Authors:** James Qian, Yu-Ling Chang, Xiaodong Liu, Joshua Goh, Angela Gutchess

**Affiliations:** Brandeis University, Waltham, Massachusetts, United States; National Taiwan University, Taipei, Taipei, Taiwan (Republic of China); Brandeis University, Waltham, Massachusetts, United States; National Taiwan University, Taipei, Taipei, Taiwan (Republic of China); Brandeis University, Waltham, Massachusetts, United States

## Abstract

Mnemonic discrimination, differentiating between similar items, declines with age. Declines may result not only from reduced hippocampal function but also from changes in executive function (EF). This study examines how memory and EF contribute to mnemonic discrimination across age and cultural groups. A total of 201 participants (98 Americans: 43 older, 55 younger; 103 Taiwanese: 50 older, 53 younger) completed the Mnemonic Similarity Task (MST) and a culturally adapted neuropsychological battery. The MST consisted of an encoding phase and a recognition phase, in which participants discriminated old items from similar and new items. Exploratory factor analysis derived memory and EF factors from the neuropsychological tests. General linear models examining the effects of memory, EF, age, culture, and two-way interactions on MST performance were significant, F(5, 179) = 21.23, p < .001, adjusted R2 = .35. Main effects of memory, age (younger), and culture (American) were observed (Ps < .01). A significant interaction between EF and age emerged, t(179) = 2.93, p < .01, such that EF correlated with MST performance in younger (p = .02) but not older adults (p = .11). These findings suggest that while memory processes support mnemonic discrimination, EF serves a compensatory role that diminishes with age, as declines in EF limit its ability to offset deficits. The results are consistent with an age-related shift toward pattern completion mechanisms. When EF can no longer compensate for declines in pattern separation, older adults rely more on pattern completion – a process for which EF is less effective.

